# Syndrome de Stewart-Treves: complication rare de lymphœdème chronique

**DOI:** 10.11604/pamj.2016.24.196.8540

**Published:** 2016-07-07

**Authors:** Sanaa Krich, Fatima Zahra Mernissi

**Affiliations:** 1 Service de Dermatologie-Vénérologie, Centre Hospitalier Universitaire Hassan II, Fès, Maroc

**Keywords:** Angiosarcome, syndrome de Stewart-Treves, complication rare de lymphœdème, Stewart-Treves syndrome, chronic lymphedema, lymphoangiosarcoma

## Image en médecine

Un patient de 43 ans, ayant une notion de paraplégie post-traumatisme avec œdème des 2 membres inférieurs (MI), consulte pour une tumeur de la jambe gauche qui remontait à 2 mois et demi. L’examen clinique objectivait une tumeur ulcéro-bourgeonnante multi-nodulaire associée à un lymphœdème bilatéral des deux MI avec des multiples nodules et plaques érythémato-violacées satellites (image A). Le diagnostic différentiel était surtout une maladie de Kaposi, mélanome achromique ou un pseudo-kaposi sur un membre paralytique. L’étude histologique avait montré une prolifération tumorale de cellules épithéloides atypiques avec un aspect fusiforme au niveau du derme. L’étude immunohistochimique notait l’expression de CD 34 et CD 31 sans expression de CK, mélan A et LCA. Le bilan d’extension avait objectivé des métastases ganglionnaires et osseuses. Le diagnostic d’angiosarcome métatstatique dans le cadre d’un syndrome de Stewart-Treves a été retenu et le malade était candidat à une amputation de propreté suivie d’une chimiothérapie palliative. L’évolution a été marquée par le décès du malade 1mois après l’amputation. Le syndrome de stewart-treves (SST) est un lymphoangiosarcome cutané compliquant un lymphœdème chronique. C’est une complication rare qui apparaît classiquement sur le bras des femmes qui ont subi une mastectomie avec curage ganglionnaire pour cancer du sein. Le traitement repose sur une exérèse large avec une radiothérapie. Cependant le risque de récidive locale et surtout de métastases (pulmonaires et osseuses) est important. Le pronostic reste mauvais avec une survie après 5 ans estimée à 10%.

**Figure 1:**
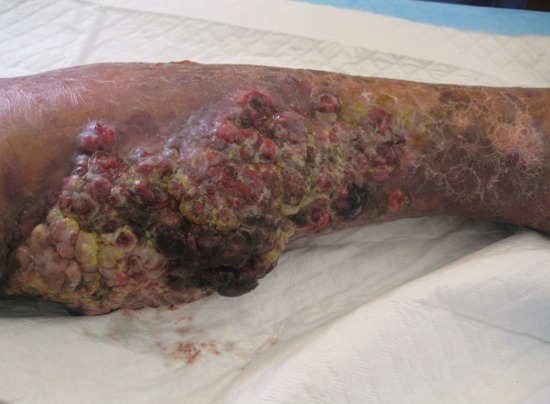
Tumeur ulcéro-bourgeonnante multi-nodulaire avec des multiples nodules et plaques érythémato-violacées satellites

